# The new era of immunotherapy for breast cancer: challenges and coping strategies of CAR-T cell therapy

**DOI:** 10.3389/fimmu.2025.1698146

**Published:** 2025-12-02

**Authors:** Zhihao Wang, Lele Miao, Wei Wang

**Affiliations:** 1 Jining Medical University, Jining, China; 2 Department of Thyroid and Breast Surgery, Jining No.1 People’s Hospital, Jining, China; 3 Medical Integration and Practice Center, Shandong University, Jinan, China

**Keywords:** breast cancer, immunotherapy, CAR-T cells, challenge, coping strategy

## Abstract

Breast cancer remains the most prevalent malignant tumor among women globally, with persistently high incidence and mortality rates. Despite the diversity of existing treatment modalities, systemic therapies for advanced, metastatic, and triple-negative breast cancer continue to face significant limitations. Recently, chimeric antigen receptor T (CAR-T) cell therapy has demonstrated considerable success in the treatment of hematological malignancies, offering a novel approach for breast cancer treatment. This paper reviews the primary targets and research advancements of CAR-T cells in breast cancer therapy, while also analyzing current challenges such as immunosuppression within the tumor microenvironment, antigen heterogeneity, obstacles in cell homing, and the limited durability of CAR-T cells. Furthermore, strategies to address these challenges are discussed. Although CAR-T cell therapy for breast cancer is still in the nascent stages of exploration, ongoing technological advancements and in-depth research hold promise for its potential as a novel therapeutic option, thereby offering renewed hope to patients.

## 1 Introduction

Breast cancer is the most prevalent malignant tumor affecting women globally, with its incidence and mortality rates remaining significantly high. Recent global cancer statistics indicate that approximately 2.3 million new cases of breast cancer are diagnosed annually, representing 11.6% of all cancer cases (1). Furthermore, breast cancer ranks first in both incidence and mortality among women (1). Currently, a variety of clinical treatments are available for breast cancer, including surgery, chemotherapy, radiotherapy, endocrine therapy, and molecular targeted therapy. Surgical resection remains the primary therapeutic approach for early-stage breast cancer; however, in cases of advanced or metastatic breast cancer, systemic treatment assumes greater importance. Chemotherapy, while widely used, is associated with systemic toxicity and the development of drug resistance. Radiotherapy is crucial for local control of breast cancer, though it carries the risk of radiation-induced damage. Endocrine therapy applies only to patients with hormone receptor-positive breast cancer. Although molecular targeted therapy has achieved some advancements for specific molecular targets, its effectiveness is constrained by issues of target selection, tumor heterogeneity, and the emergence of drug resistance (2). Similarly, immune checkpoint inhibitors (ICIs) have shown effectiveness in a subset of patients; however, their success is constrained by the expression levels of immune checkpoints and the intricate nature of the tumor microenvironment. Furthermore, treatment options for triple-negative breast cancer (TNBC) are particularly restricted due to the absence of effective therapeutic targets, resulting in poorer patient prognoses (3). These challenges highlight the critical need for the development of novel treatment strategies.

**Figure 1:**
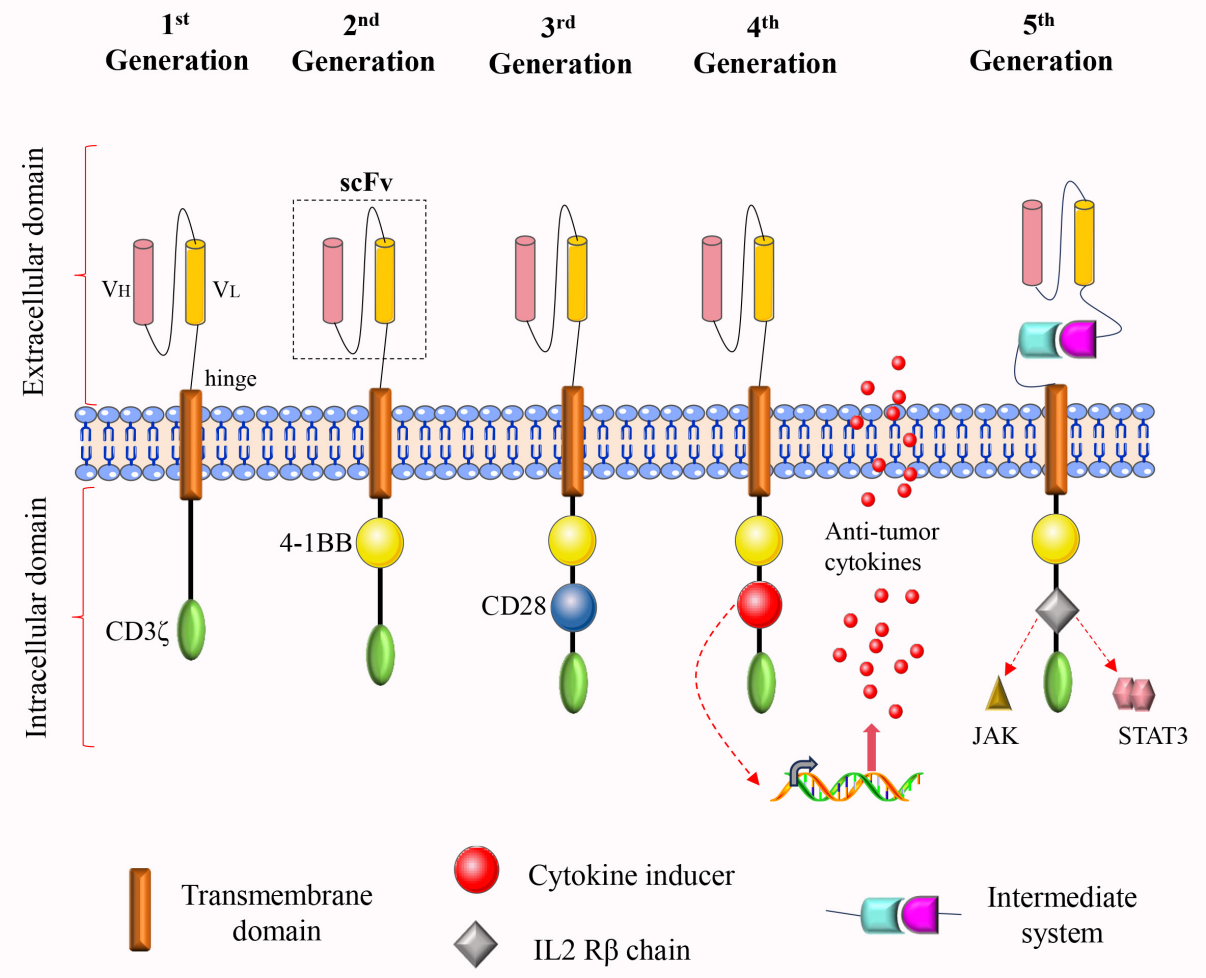
The structural evolution of CAR-T cells from the first to the fifth generation. The intracellular domain of the first-generation CAR comprises solely the CD3ζ activation domain, which is inadequate for effective proliferation and persistence. To address this limitation, a costimulatory molecule was incorporated into the intracellular domain of the second-generation CAR, thereby enhancing both proliferation and durability. The third-generation CAR further augments anti-tumor activity by incorporating two costimulatory molecules within its intracellular domain. In the fourth-generation CAR, either a cytokine secretion module or a suicide gene is introduced into the intracellular domain to enhance functionality. The fifth-generation CAR integrates an IL-2Rβ fragment within its intracellular domain, thereby activating the JAK-STAT signaling pathway. Additionally, an intermediate system may be incorporated into the extracellular domain, allowing for the flexible identification of various tumor antigens through the replacement of different antigen-binding modules.

The introduction of CAR-T cell therapy has marked a transformative advancement in the management of malignant tumors. Since the United States Food and Drug Administration (FDA) approved CAR-T cell therapy in the treatment of relapsed/refractory B-cell acute lymphoblastic leukemia (B-ALL) in 2017, CAR-T therapy has achieved significant milestones in the treatment of hematological malignancies. These successes offer valuable insights for the potential treatment of solid tumors. In recent years, CAR-T cell research has achieved rapid advancements, evolving from the first to the fifth generation (**Figure 1**). In the domain of breast cancer treatment, CAR-T cell therapy has transitioned from a “proof of concept” phase to an “early clinical development stage”. Researchers are actively investigating a range of CAR-T cell therapies targeting breast cancer-related antigens. Nonetheless, this therapeutic approach encounters several significant challenges in the treatment of breast cancer (4). Firstly, the immunosuppressive nature of the tumor microenvironment hinders the efficacy of CAR-T cells at tumor sites. Secondly, antigen heterogeneity poses a risk, as it may prevent CAR-T cells from recognizing all cancer cells, thereby increasing the likelihood of tumor recurrence. Thirdly, the homing challenge of CAR-T cells complicates their precise delivery to tumor sites. Fourthly, the limited persistence of CAR-T cells constrains their long-term anti-tumor efficacy. Lastly, toxic reactions may arise during treatment, impacting patient safety and tolerance (5). Furthermore, the field also faces several practical problems: a) The efficacy of CAR-T cell clinical trials in breast cancer remains generally limited, and the successes observed in the treatment of hematological malignancies have not been replicated; b) The field has suffered major safety setbacks, especially the fatal acute lung injury in the early HER2-CAR-T cell test (6); c) CAR-T cell therapy for breast cancer also encounters competition from emerging immunotherapies, which may influence its clinical positioning.

This review aims to elucidate the research context and deepen the understanding of the opportunities and challenges associated with CAR-T therapy in the treatment of breast cancer. It is intended to serve as a valuable reference for future basic research, translational exploration, and clinical trial design. By providing a comprehensive summary and in-depth analysis of existing research findings, this review aspires to advance the application of this promising therapy, ultimately benefiting a broader population of breast cancer patients.

## 2 The main target of CAR-T cell therapy for breast cancer

### 2.1 Receptor tyrosine kinase targets

Receptor tyrosine kinases (RTKs) are integral to cellular signal transduction, and their dysregulated expression is associated with the initiation and progression of various cancers. Consequently, RTK targets have emerged as significant targets for CAR-T cell therapy in the treatment of breast cancer (7). For instance, HER2-targeting CAR-T cell therapy has exhibited potent cytotoxicity toward HER2-positive breast cancer cells in preclinical studies, thereby providing a novel therapeutic strategy for patients with this subtype (8). Additionally, the overexpression of EGFR in breast cancer correlates with tumor progression and poor prognosis, making it a critical target for CAR-T cell therapy. CAR-T cells engineered to target EGFR have exhibited strong and specific inhibitory effects on TNBC cells both *in vitro* and *in vivo* (9). Furthermore, ROR1 (10) and c-Met (11) are also classified as PTK targets. A comprehensive investigation into these RTK targets is essential for the development of more efficacious CAR-T cell therapies.

### 2.2 Cell surface glycoproteins targets

Cell surface glycoproteins are crucial for the recognition and elimination of tumor cells, serving as specific targets for CAR-T cell therapy in breast cancer. MUC1 is notably overexpressed in early basal-like triple-negative breast cancer, which provides a theoretical basis for MUC1-based immunotherapy (12). MUC1-CAR-T cells have demonstrated significant target-specific cytotoxicity both *in vivo* and *in vitro*, effectively inhibiting tumor growth (13). Trop2, a transmembrane glycoprotein, is widely expressed in various solid tumors, particularly in TNBC (14). The potential of Trop2 as a target in CAR-T cell therapy has been validated. For instance, a recent study has demonstrated that Trop2-targeting fourth-generation CAR-T cells display robust anti-tumor activity both *in vitro* and *in vivo*, effectively eliminating Trop2-positive breast cancer cells (15). Additionally, MSLN (16) and ICAM1 (17) are also identified as cell surface glycoprotein targets. **Table 1** provides a summary of common CAR-T cell therapy targets for breast cancer.

**Table 1:** Common targets of CAR-T cells for the treatment of breast cancer.

Target	Phase	CAR design	The main research contents and results	References
c-Met	Phase 0 (6 patients)	2nd generation CAR	Six patients with metastatic breast cancer were injected with c-Met-CAR T cells. The results showed that significant tumor necrosis and immune response were caused at the injection site; the patients were well tolerated, and no serious adverse reactions occurred.	(11)
MUC1	Preclinical research	4th generation CAR; releasing IL-12	The researchers constructed a novel MUC1-CAR-T cell by introducing multiple modifications, such as PD1 ^KO^, IL-12 release, and TGFBR2 ^KO^. The results showed that these CAR-T cells could not only play a powerful role in the local tumor microenvironment but also control distant metastases effectively.	(18)
ZP4	Preclinical research	2nd generation CAR	Through comprehensive bioinformatics analysis, researchers found that ZP4 could be used as a new target of TNBC and generated monoclonal antibodies against ZP4 to construct CAR-T cells. The experimental results showed that ZP4-CAR-T cells could effectively kill ZP4-positive TNBC cells *in vitro* and *in vivo*.	(19)
MSLN	Preclinical research	4th generation CAR; releasing NKG2D- BiTEs	Researchers developed a new type of Bispecific T cell engagers CAR-T (BiTEs CAR-T) cells, which can target MSLN and secrete NKG2D-BiTEs to bind NKG2D ligands. The results showed that these BiTEs CAR-T cells exhibited excellent performance in killing tumor cells, activating T cells, and producing cytokines. Moreover, they demonstrated a remarkable anti-tumor effect and good safety profile in animal models.	(16)
EGFR	Preclinical research	2nd generation CAR	The combination of EGFR-CAR-T cell therapy and radiotherapy showed an enhanced anti-tumor effect in both the *in situ* TNBC mouse model with normal immune function and that with immunodeficiency. This combination therapy not only enhanced the infiltration of CAR-T cells in the tumor, but also did not increase the risk of CRS.	(20)
ROR1	Preclinical research	4th generation CAR; releasing anti-PD-1 scFv	Researchers developed a novel ROR1-CAR-T cell engineered to release anti-PD-1 scFv upon activation, which enhanced the activity of both the CAR-T cells and TILs. Both *in vitro* and *in vivo* experiments showed that these CAR-T cells significantly improved the therapeutic efficacy against TNBC, prolonged the survival time of mice, and increased the number of tumor-infiltrating T cells.	(10)
Trop2	Preclinical research	Atypical 4th generation CAR	Researchers developed a novel fourth-generation CAR (CAR4) incorporating three costimulatory domains (CD28, 4-1BB, and CD27) fused to CD3ζ. Results demonstrated that these CAR-T cells not only enhanced cytotoxicity and IFN-γ production against Trop2-positive breast cancer cells but also conferred better long-term persistence and proliferative capacity.	(15)
tmTNF-α	Preclinical research	2nd generation CAR	This study investigated the efficacy of CAR-T cells targeting tmTNF-α against breast cancer. The results demonstrated that tmTNF-α-CAR-T cells effectively killed tmTNF-α-positive tumor cells and inhibited tumor growth. Furthermore, blocking the PD-1/PD-L1 pathway synergized with and enhanced the anti-tumor efficacy of tmTNF-α CAR-T cell therapy.	(21)
GD2	Preclinical research	2nd generation CAR	In the TNBC xenotransplantation model, GD2-CAR-T cells not only significantly inhibited primary tumor growth but also prevented lung metastasis.	(22)
HER2	Preclinical research	2nd generation CAR	HER2-CAR-T cells could effectively guide immune effector cells to recognize and kill HER2-positive tumor cells and inhibit tumors in model mice.	(8)
CD24	Preclinical research	2nd generation CAR	CD24-CAR-T cells had strong anti-tumor activity against CD24-positive breast cancer cells both *in vivo* and *in vitro*.	(23)

TME, tumor microenvironment.

TNBC, triple-negative breast cancer.

MSLN, mesothelin.

CRS, cytokine release syndrome.

scFv, single-chain variable fragments.

TILs, tumor-infiltrating lymphocytes.

### 2.3 Related clinical trials

Currently, clinical trials investigating CAR-T cell therapy for breast cancer are actively underway to assess its safety and efficacy. Nevertheless, this area of research remains in its nascent stages, resulting in a limited number of clinical trials. We summarized some relevant clinical trials for breast cancer (**Table 2**). The progression of these trials is anticipated to yield valuable practical insights into the application of CAR-T cells for breast cancer treatment and facilitate the translation of this therapy into clinical practice.

**Table 2:** Clinical trials related to CAR-T cell therapy for breast cancer.

No.	Study title	Targets	Study start	Phase	Enrollment	Locations	Status	NCT Number	Key outcomes/findings
1	Chimeric Antigen Receptor-Modified T Cells for Breast Cancer	HER2	2015-09	Phase 1 Phase 2	Unknown	Central laboratory in Fuda cancer hospital, Guangzhou, Guangdong, China	Withdrawn	NCT02547961	No Results Posted
2	Treating Nectin-4-positive Advanced Breast Cancer with XKDCT293 (Nectin-4-CAR-T)	Nectin-4	2024-03	Phase 1	18	AnYang Tumor Hospital, Anyang, Henan, China	Recruiting	NCT06724835	No Results Posted
3	PD-1 Knockout Anti-MUC1 CAR-T Cells in the Treatment of Advanced Breast Cancer	MUC1	2019-05	Phase 1 Phase 2	15	Sun Yat-sen Memorial Hospital of Sun Yat-sen University, Guangzhou, Guangdong, China	Completed	NCT05812326	No Results Posted
4	Autologous huMNC2-CAR44 or huMNC2-CAR22 T Cells for Breast Cancer Targeting Cleaved Form of MUC1 (MUC1*)	MUC1	2020-01	Phase 1	69	City of Hope Medical Center, Duarte, California, United States	Recruiting	NCT04020575	No Results Posted
5	EGFR/B7H3 CAR-T on Lung Cancer and Triple Negative Breast Cancer	EGFR B7H3	2022-05	Early Phase 1	30	Second Affiliated Hospital of Guangzhou Medical University, Guangzhou, Guangdong, China	Recruiting	NCT05341492	No Results Posted
6	CAR T Cells in Mesothelin-Expressing Breast Cancer	Mesothelin (MSLN)	2023-02	Phase 1	2	University of Pennsylvania, Philadelphia, Pennsylvania, United States	Terminated	NCT05623488	No Results Posted
7	TRAIL-R2 and HER2 Bi-Specific Chimeric Antigen Receptor (CAR) T Cells for the Treatment of Metastatic Breast Cancer	TRAIL-R2 HER2	2025-09	Phase 1	27	Houston Methodist Hospital, Houston, Texas, United States	Not yet recruiting	NCT06251544	No Results Posted
8	Multi-4SCAR-T Therapy Targeting Breast Cancer	HER2 GD2 CD44v6	2020-06	Phase 1 Phase 2	100	The Seventh Affiliated Hospital, Sun Yat-Sen University, Shenzhen, Guangdong, China	Unknown status	NCT04430595	No Results Posted
9	A Study to Assess CLBR001+ABBV-461 in Subjects with Locally Advanced or Metastatic Breast Cancer	Unknown	2025-04	Phase 1	20	Indiana University Melvin and Bren Simon Comprehensive Cancer Center, Indianapolis, Indiana, United States Roswell Park Cancer Institute, Buffalo, New York, United States University of Virginia, Charlottesville, Virginia, United States	Active, not recruiting	NCT06878248	No Results Posted
10	c-Met CAR RNA T Cells Targeting Breast Cancer	c-Met	2013-04	Phase 1	6	Abramson Cancer Center of the University of Pennsylvania, Philadelphia, Pennsylvania, United States	Completed	NCT01837602	Intratumoral injections of mRNA c-Met-CAR T cells are well tolerated and evoke an inflammatory response within tumors (11).
11	T-Cell Therapy for Advanced Breast Cancer	Mesothelin (MSLN)	2016-06	Phase 1	186	Memorial Sloan Kettering Cancer Center (Consent and follow-up only), Basking Ridge, New Jersey, United States Memorial Sloan Kettering Monmouth (Consent and follow-up only), Middletown, New Jersey, United States Memorial Sloan Kettering Bergen (Consent and follow-up only), Montvale, New Jersey, United States	Active, not recruiting	NCT02792114	No Results Posted
12	Study of Autologous CAR-T Cells Targeting B7-H3 in TNBC iC9-CAR.B7-H3 T Cells	B7-H3	2024-06	Phase 1	42	University of North Carolina, Chapel Hill, North Carolina, United States	Recruiting	NCT06347068	No Results Posted
13	Genetically Modified T-Cell Therapy in Treating Patients With Advanced ROR1+ Malignancies	ROR1	2016-03	Phase 1	21	Seattle, Washington, United States	Terminated	NCT02706392	Two of the three (67%) patients with chronic lymphocytic leukemia showed robust CAR-T-cell expansion and a rapid antitumor response. In patients with non-small cell lung cancer and triple-negative breast cancer, CAR-T cells expanded to variable levels and infiltrated tumors poorly and 1 of 18 patients (5.5%) achieved partial response (24).
14	Autologous T Cells Expressing MET scFv CAR (RNA CART-cMET)	c-Met	2016-12	Early Phase 1	77	Philadelphia, Pennsylvania, United States	Terminated	NCT03060356	Intravenous RNA-electroporated cMET-CAR T cells induced only grade 1–2 toxicities without ≥ grade 3 adverse events or neurotoxicity in seven patients, confirming the regimen’s safety and feasibility. In terms of curative effect, 4 patients were stable and 3 patients were progressive (25).

Nevertheless, current evidence from published clinical trial results indicates that the overall efficacy of CAR-T cell therapy in breast cancer remains limited, especially when compared to its notable success in treating hematological malignancies. For instance, in a Phase 1 clinical trial (25), four patients with metastatic melanoma and three patients with metastatic TNBC were administered intravenous injections of RNA-electroplated cMET-CAR-T cells. While the trial demonstrated manageable safety profiles, the anti-tumor efficacy was constrained, with four patients maintaining stable disease and three experiencing disease progression. Similarly, in another Phase 1 clinical trial (24), involving 18 patients with solid tumors (comprising 10 patients with TNBC and 8 with non-small cell lung cancer), treatment with ROR1-CAR-T cells resulted in only one patient achieving partial remission. These findings underscore the need for further enhancement of CAR-T cell therapy efficacy in the clinical management of breast cancer.

## 3 The key challenge of CAR-T cells in the treatment of breast cancer

While CAR-T cells hold promise for the treatment of breast cancer, they encounter numerous challenges that limit their efficacy and broader application. These challenges primarily encompass the lack of suitable target antigens, the TME, antigen heterogeneity, obstacles related to homing, limited persistence, toxic reactions, and high costs. Addressing these issues is crucial for enhancing the therapeutic effectiveness of CAR-T cells in breast cancer treatment.

### 3.1 Lack of suitable target antigens

The identification of safe and effective target antigens is a critical prerequisite for successful CAR-T cell therapy. However, breast cancer currently lacks optimal candidates that fully satisfy clinical requirements. An ideal tumor antigen for CAR-T cell therapy should meet three fundamental criteria: a) High and specific expression on tumor cells. The antigen should be abundantly expressed on breast cancer cells to ensure that CAR-T cells can specifically recognize and eliminate malignant cells; b) Minimal or absent expression on normal tissues. It is crucial that the antigen exhibits low or no expression on healthy cells to prevent the “on-target off-tumor effect,” a significant cause of treatment-related toxicity, where CAR-T cells may inadvertently attack normal cells expressing the target antigen; c) Stable expression. The antigen should maintain consistent expression and not be easily downregulated or lost due to tumor evolution, as such changes could lead to immune escape and therapeutic failure.

In contemporary research on CAR-T cell therapy for breast cancer, the predominant focus is on tumor-associated antigens (TAAs) rather than tumor-specific antigens (TSAs). TAAs, including HER2, EGFR, and Trop2, are characterized by their overexpression in breast cancer tissues, yet they also demonstrate low-level expression in normal tissues, thereby introducing inherent safety risks. Conversely, TSAs are exclusively expressed by tumor cells, rendering them theoretically ideal targets for CAR-T cell therapy. Nonetheless, the significant interpatient and intratumor heterogeneity observed in breast cancer presents substantial challenges in the screening, validation, and personalized application of TSAs. These challenges are both technically demanding and financially burdensome, thereby impeding their translation into clinical practice. The lack of TSAs has become an important bottleneck in this research field.

#### 3.1.1 Coping strategies

In recent years, advancements in genomics and bioinformatics technologies have facilitated the identification and utilization of TSAs. These methodologies not only aid in the identification of TSAs but also provide novel targets for CAR-T cell therapy, thereby enhancing its efficacy in the treatment of solid tumors. Currently, in-depth exploration using multi-omics and bioinformatics approaches is an effective strategy for discovering authentic TSAs. Through high-throughput sequencing analysis of the genome and transcriptome of tumor tissues, novel antigens, known as neoantigens, arising from somatic mutations, including point mutations and frameshift mutations, can be identified (26). These neoantigens are encoded by tumor-specific mutations and have the potential to elicit individualized immune responses, making them ideal candidates for TSAs (27). Furthermore, emerging artificial intelligence algorithms can predict neoantigens generated by non-mutational mechanisms, such as aberrant RNA splicing, from large datasets. These algorithms enable a systematic analysis of the expression profiles of these antigens in both tumor and normal tissues, thereby facilitating the identification of TSAs with high specificity and broad coverage (26, 28).

### 3.2 Immunosuppression of TME

The TME of solid tumors constitutes a complex and dynamic ecosystem. It encompasses not only tumor cells but also a variety of immune cells, stromal cells, and the extracellular matrix, all of which collectively influence tumor growth and metastasis (29).

Within the TME, key inhibitory cell populations include myeloid-derived suppressor cells (MDSCs), tumor-associated macrophages (TAMs), regulatory T cells (Tregs), and cancer-associated fibroblasts (CAFs). MDSCs suppress T-cell function and metabolism through two primary mechanisms: the depletion of essential nutrients, including arginine and cysteine, and the secretion of reactive oxygen species (ROS) and nitric oxide (NO), which are directly toxic to T cells (30). TAMs can undergo polarization to the M2 phenotype, which exerts immunosuppressive effects by secreting cytokines such as TGF-β and IL-10, thereby inhibiting T cell proliferation and activation (31). Tregs suppress the anti-tumor activity of CAR-T cells through direct cell-to-cell contact and the secretion of inhibitory cytokines, including IL-10 and TGF-β (32). CAFs contribute to the formation of a physical barrier by secreting cytokines and remodeling the extracellular matrix (ECM), which obstructs the infiltration and function of CAR-T cells (33, 34).

**Figure 2:**
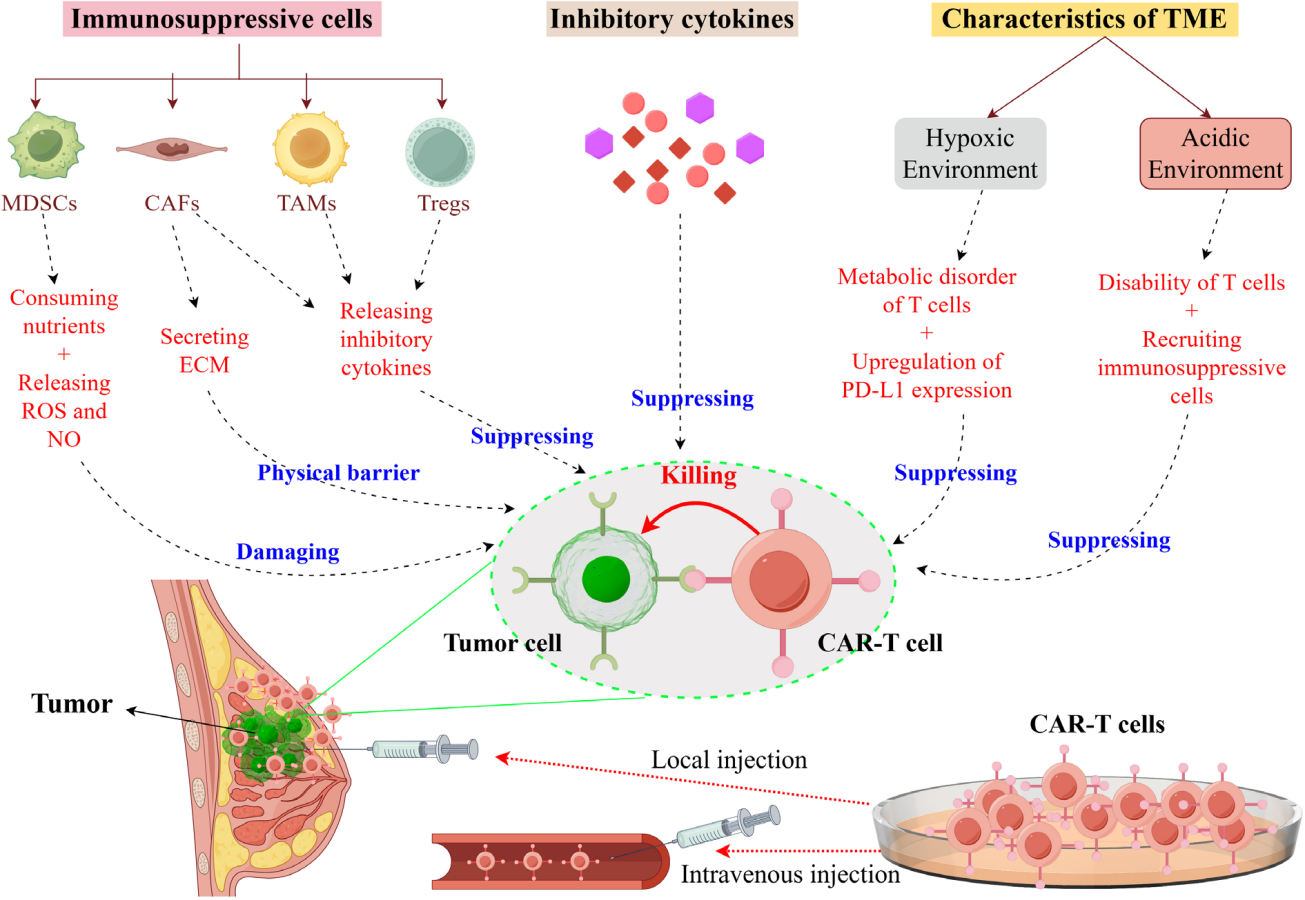
Immunosuppressive mechanisms of the TME in breast cancer on CAR-T Cells. It illustrates the immunosuppressive mechanisms exerted by the TME in breast cancer, which compromise the functionality of CAR-T cells. Within the TME, immunosuppressive cells such as myeloid-derived suppressor cells (MDSCs), cancer-associated fibroblasts (CAFs), tumor-associated macrophages (TAMs), and regulatory T cells (Tregs) contribute to this suppression by depleting essential nutrients and secreting both the extracellular matrix (ECM) and inhibitory cytokines. These cytokines directly inhibit the cytotoxic activity of CAR-T cells. Furthermore, the hypoxic conditions within the TME lead to metabolic dysregulation in T cells and an upregulation of programmed death-ligand 1 (PD-L1) expression, while the acidic environment results in T cell dysfunction and the recruitment of additional immunosuppressive cells. Collectively, these factors diminish the efficacy of CAR-T cells in targeting tumor cells. This figure was drawn by Figdraw (a drawing platform).

Hypoxic and acidic conditions within the TME impose adverse effects on CAR-T cell function and persistence (35, 36). Hypoxic conditions disrupt T cell metabolism, impairing their proliferation and survival, while also inducing tumor cells to express increased levels of immunosuppressive molecules, such as PD-L1 (35). The acidic milieu of the TME further compromises the functionality of tumor-infiltrating T cells, leading to a state of dysfunction characterized by reduced cytolytic activity and diminished cytokine secretion (37). Additionally, the acidic environment facilitates the recruitment and activation of immunosuppressive cells, thereby exacerbating immunosuppression (38). The TME also harbors a plethora of inhibitory cytokines, including IL-4 and TGF-β. IL-4, an inhibitory cytokine, impairs the effector function of T cells via binding to IL-4 receptors on T cell surfaces (39). TGF-β, a pivotal inhibitory cytokine within the TME, impairs the persistence and functionality of effector T cells by interacting with receptors on Tregs, consequently attenuating the anti-tumor immune response (40). **Figure 2** provides a summary and depiction of the impact of key inhibitory factors in the TME, like inhibitory cells, cytokines, and the physicochemical microenvironment, on CAR-T cell functions.

#### 3.2.1 Coping strategies

The targeted elimination of immunosuppressive cells within the TME or the inhibition of their functions represents an important strategy for enhancing the efficacy of CAR T-cell therapy. For instance, MDSCs have been shown to impair the function and persistence of CAR-T cells (41). One study investigated the enhancement of CAR-T cell therapy efficacy in breast cancer treatment by selectively targeting the TRAIL receptor 2 (TR2) on MDSCs. Researchers developed a CAR-T cell targeting MUC1, which co-expresses a novel costimulatory receptor capable of targeting TR2 on MDSCs. The findings indicate that this bifunctional CAR T cell not only directly eradicates tumor cells but also restores and enhances its function and durability within the TME by inducing apoptosis in MDSCs, thereby significantly improving therapeutic outcomes (42). Moreover, another study (43) demonstrated that Olaparib, a PARP inhibitor, significantly augmented the anti-tumor efficacy of EGFRvIII-CAR-T cells in a murine breast cancer model by inhibiting MDSC migration mediated by the SDF1α/CXCR4 axis. This discovery not only reveals the new mechanism of PARP inhibitors but also provides theoretical support and potential clinical application direction for the combined treatment of breast cancer.

To address the suppressive impact of inhibitory cytokines, such as IL-4 and TGF-β, within the TME, researchers have developed an inverted cytokine receptor (ICR) designed to convert inhibitory signals into stimulatory ones. This innovation aims to enhance the viability and anti-tumor efficacy of CAR-T cells within the TME (39, 44). For instance, Bajgain et al. (45) engineered MUC1-CAR-T cells to co-express the inverted cytokine receptor (4/7ICR), thereby converting the inhibitory IL-4 signal ubiquitous in TME into an activation signal. The findings demonstrated that MUC1-CAR-T cells expressing 4/7ICR were capable of effectively and durably eradicating breast cancer cells both *in vitro* and *in vivo*. Furthermore, the efficacy of CAR-T cells can be enhanced by inhibiting the signaling pathways associated with these inhibitory cytokines. Stüber et al. (46) demonstrated that TGF-β significantly suppressed the anti-tumor activity of ROR1-CAR-T cells in TNBC, as evidenced by reduced cytotoxicity, diminished cytokine secretion, impaired proliferative capacity, and decreased survival rates. The application of SD-208, a TGF-β receptor signaling pathway inhibitor, effectively safeguarded ROR1-CAR-T cells, counteracted the suppressive effects of TGF-β, and preserved their anti-tumor functionality in both *in vitro* and three-dimensional tumor models.

Transforming CAR-T cells into “regulators” of TME. For instance, the fourth-generation CAR-T cells that can secrete cytokines such as IL-10, IL-12, or IL-15 has been shown to enhance their antitumor efficacy in solid tumors and improve their functionality within the TME (47–49). Furthermore, Erler et al. (18) developed a multi-armed allogeneic MUC1-CAR-T cell therapy for TNBC. To overcome the obstacles of the TME in TNBC, the researchers employed transcription activator-like effector nuclease (TALEN) technology for multi-layer gene editing of the CAR-T cells. This approach endowed the cells with PD-1 knockout, TGFBR2 knockout, and IL-12 secretion capabilities. The findings indicated that this innovative design significantly enhanced the antitumor activity of CAR-T cells and effectively reduced both local and distant tumor burdens in a TME enriched with TGFB1 and PD-L1, all while maintaining treatment safety.

To enhance the efficacy of CAR-T cells, it is imperative to implement strategies addressing the hypoxic and acidic conditions of the TME. For instance, Zhu et al. (50) developed hypoxia-responsive CAR-T cells by incorporating hypoxia response elements and oxygen-dependent degradation domains, ultimately identifying 51p-CEA-CAR-T cells that demonstrated both safety and efficacy. Their findings indicated that these hypoxia-responsive CAR-T cells could sustain a low level of differentiation, augment oxidative metabolism and proliferation, and mitigate the adverse effects of hypoxia on T cells. Furthermore, *in vivo* experiments revealed that these CAR-T cells exhibited reduced T cell exhaustion, improved T cell phenotype, and prolonged anti-tumor activity. The acidic microenvironment is one of the important factors that inhibit the function of CAR-T cells. Research by Zhai et al. (51) demonstrated that Pantoprazole, a proton pump inhibitor frequently employed in clinical practice, can enhance the expression of the MSLN protein by mitigating the acidic TME inTNBC brain metastases through an increase in TME pH. This modulation subsequently augmented the efficacy of MSLN-CAR-T cell therapy. This finding introduced a novel immunotherapeutic strategy for the treatment of solid tumors with brain metastases, particularly in cases of TNBC and non-small cell lung cancer with brain metastases.

The integration of nanotechnology with CAR-T cell therapy has led to significant advancements in enhancing its functional performance within the TME. Nano-catalysts can improve the activation and infiltration of CAR-T cells by disrupting the dense structure of tumors, thereby augmenting their therapeutic efficacy (52). Furthermore, surface engineering through nanotechnology can further enhance CAR-T cell function. By encapsulating drugs within nanoparticles and attaching them to the surface of CAR-T cells, inhibitory molecular antagonists can be effectively delivered to tumor-infiltrating lymphocytes, thereby mitigating the immunosuppressive effects of the TME and enhancing the anti-tumor activity of CAR-T cells (53). In conclusion, the application of nanotechnology offers a novel strategy to address these challenges.

#### 3.2.2 The critical assessment of coping strategies

The current strategies for addressing immunosuppression within the TME are innovative and hold promise for clinical application. Nonetheless, these strategies encounter several practical challenges during clinical translation:

a) Although strategies targeting suppressive cells can directly remodel TME, there are multiple immunosuppressive cells in TME, and a single targeting strategy may be ineffective due to compensatory mechanisms.

b) Transforming inhibitory cytokine signals into stimulatory signals aims to enhance the activity and antitumor efficacy of CAR-T cells (54). However, this approach carries the risk of excessive activation and uncontrolled proliferation of CAR-T cells *in vivo*, potentially resulting in immune-related adverse events.

c) Approaches aimed at enhancing the physical and chemical properties of the TME, such as the development of hypoxia-responsive CAR-T cells, focus more on conferring cellular “tolerance” to adverse conditions rather than directly eliminating hypoxia. The complexity involved in the construction of these CAR-T cells may impede their large-scale production and clinical application.

d) While nanotechnology offers novel avenues for augmenting the functionality of CAR-T cells within the TME, issues related to the biological safety, scalability, and feasibility of large-scale nanomaterial production remain significant obstacles in clinical translation (55). Currently, the application of nanotechnology in CAR-T cell therapy is in the early stage, with a paucity of data from large-scale clinical trials.

### 3.3 Antigen heterogeneity

Breast cancer is characterized by pronounced antigen heterogeneity, indicating substantial variability in antigen expression among tumor cells across different regions, lesions, or treatment stages within the same tumor. This heterogeneity poses significant challenges to CAR-T cell therapy. Tumor cells can evade CAR-T cell-mediated cytotoxicity by down-regulating or losing specific antigens, a phenomenon that significantly contributes to therapeutic failure (5, 56). For instance, in HER2-positive breast cancer, certain tumor cells may exhibit reduced HER2 expression due to epigenetic modifications, rendering HER2-CAR-T cells ineffective in recognizing and eliminating these cells. Furthermore, the antigenic diversity complicates the ability of CAR-T cells to comprehensively target and eradicate all cancer cells, thereby elevating the risk of tumor recurrence (57). In patients with breast cancer, there exists heterogeneity in antigen expression among cancer cells, with some exhibiting high levels of expression while others demonstrate low or absent expression. As a result, CAR-T cells, which are designed to target cells with high antigen expression, may fail to recognize and eliminate those with low or absent expression. This limitation can facilitate the evasion of immune detection by certain cancer cells, thereby increasing the risk of tumor recurrence.

#### 3.3.1 Coping strategies

One of the strategies to overcome this challenge is to enhance the diversity of antigen recognition by designing a logic gate control system. Notably, OR-gate dual-target or multi-target CAR-T cells demonstrate distinct advantages. These CAR constructs incorporate multiple antigen-binding domains, enabling the activation of CAR-T cell cytotoxic functions upon recognition of any single antigen. This OR-gate logic paradigm overcomes the limitations inherent in traditional single-target CARs by accommodating various antigen expression profiles without necessitating the simultaneous expression of all targets by tumor cells. Consequently, this approach substantially mitigates the risk of immune evasion due to the loss of a single antigen.

Another practical strategy is sequential combined therapy of two single-target CAR-T cells. The advantages of this method mainly focus on accuracy, safety, and practicality. Initially, this approach allows for the specific targeting of tumor antigens. CAR-T cells directed against the dominant antigen are employed to eliminate the primary tumor burden, followed by the use of CAR-T cells targeting secondary antigens to eradicate any residual subsets that are negative for the dominant antigen. This strategy aims to prevent recurrence due to antigen escape, which can occur with single-target therapies. Secondly, this method enhances safety. Single-target CAR-T cells enable independent dose optimization, allowing for adjustments based on therapeutic responses observed in earlier stages of sequential infusion, thereby mitigating associated risks. Lastly, the approach is practical. The development of single-target CAR-T cells is well-advanced, with substantial clinical data available. This eliminates the need to redesign multi-target CARs, allowing for the flexible combination of existing products based on the patient’s antigen profile. Consequently, this reduces the transition period and facilitates the implementation of personalized treatment strategies.

The SUPRA CAR system exemplifies a universal chimeric antigen receptor (CAR) framework that enables dynamic modulation of antigen specificity in CAR-T cells through the use of exogenous antibody bridging molecules (58). This innovative design enables the universal CAR to direct T cells against a diverse array of tumor antigens without necessitating additional genetic modifications, thereby enhancing the treatment’s flexibility and adaptability.

#### 3.3.2 The critical assessment of coping strategies

Antigenic heterogeneity represents a significant challenge in CAR-T cell therapy for solid tumors. Despite the promise shown by current strategies to address this issue, notable limitations persist. A multi-target approach, which involves targeting multiple antigens, offers a potential solution to heterogeneity; however, it must navigate the trade-off between complexity and safety. For instance, OR-gate dual-target CAR-T cells can effectively mitigate the risk of single antigen escape, yet their construction and production are more intricate, potentially increasing the likelihood of “off-target” toxicity in normal tissues. Similarly, the sequential combination therapy involving two single-target CAR-T cells can decrease the risk of escape through successive rounds of antigen targeting, but it also presents challenges. Specifically, the necessity to independently prepare two distinct CAR-T cell products substantially elevates production costs and imposes a greater economic burden on patients. Furthermore, the complexity of treatment is increasing. It is essential to carefully coordinate the treatment sequence, dosage, and timing intervals for the administration of two types of CAR-T cells. Any minor deviation in treatment protocol may compromise therapeutic efficacy and potentially lead to adverse reactions. Finally, the development and implementation of allogeneic CAR-T cell therapy present significant challenges. While allogeneic CAR-T cell therapy is proposed as a solution to the prolonged manufacturing times and high costs associated with autologous CAR-T cell therapy, it still carries risks of graft-versus-host disease (GVHD) and host-versus-graft reaction (HVGR) (59). Although gene editing technologies have the potential to mitigate these risks, they may introduce new issues, such as genotoxicity (59).

### 3.4 CAR-T cell homing disorder

The impaired homing ability of CAR-T cells poses a substantial limitation to their therapeutic effectiveness in the treatment of breast cancer. Firstly, tumor vascular abnormality is an important factor affecting the homing of CAR-T cells. Research indicates that the non-responsive nature of tumor blood vessels, coupled with low expression levels of leukocyte adhesion molecules, restricts the effective infiltration of CAR-T cells (60). Secondly, the physical barrier represents a significant factor contributing to the impediment of CAR-T cell homing. Within the breast cancer TME, cancer-associated fibroblasts (CAFs) undergo proliferation and secrete collagen and fibronectin, resulting in the formation of a dense extracellular matrix (ECM). This ECM constitutes a physical barrier that hinders the infiltration of CAR-T cells into the tumor core (61, 62). Thirdly, the mismatch between chemokines and their corresponding receptors constitutes a significant factor contributing to the diminished homing efficiency of T cells (63). Chemokines, which are small-molecule proteins, are crucial for regulating the migration and localization of immune cells within tissues. The interaction between chemokines and their receptors is pivotal in the TME, influencing both the recruitment of immune cells and the mechanisms of immune evasion employed by tumors (64). A mismatch between chemokines and chemokine receptors results in inadequate infiltration of effector T cells into solid tumors, thereby diminishing the efficacy of anti-tumor responses (65).

#### 3.4.1 Coping strategies

An effective local drug delivery strategy can precisely target CAR-T cells to tumor sites, thereby enhancing their capacity to overcome physical barriers. The metastasis of breast cancer to the brain remains a significant challenge in clinical treatment. The efficacy of CAR-T cells in treating brain metastases is limited due to the difficulty in fully traversing the blood-brain barrier. In a study conducted by Priceman et al. (66), the effectiveness of HER2-CAR T cell delivery was assessed using three different methods: intravenous injection, intratumoral local injection, and intraventricular injection, within a mouse model of breast cancer brain metastasis. The results demonstrated that local and intraventricular injections of HER2-CAR T cells into the brain exhibited significant anti-tumor activity in the xenotransplantation model.

To enhance the penetration and migratory capacity of CAR-T cells within solid tumors, it is essential to optimize their structural attributes. Solid tumors are characterized by a dense extracellular matrix (ECM), predominantly composed of heparan sulfate proteoglycans (HSPGs), which impede T cell infiltration. Caruana et al. (67) engineered CAR-T cells to express heparanase (HPSE), resulting in a marked improvement in the penetration and homing capabilities of CAR-T cells within tumors by degrading HSPGs. This strategy presents a novel approach for increasing CAR-T cell infiltration in solid tumors. Furthermore, by genetically modifying CAR-T cells to overexpress specific chemokine receptors, such as CCR6 (68), CXCR5 (68), and CXCR6 (69), their migration ability to tumor sites can be enhanced.

#### 3.4.2 The critical assessment of coping strategies

Although the coping strategies for addressing the homing disorder of CAR-T cells demonstrate potential, they also reveal certain limitations. Firstly, local administration can circumvent the systemic circulation and deliver CAR-T cells directly to the tumor site, thereby significantly enhancing the local concentration of CAR-T cells. However, in the case of extensively metastatic tumors, local administration struggles to encompass all tumor lesions, thereby constraining its therapeutic efficacy. Secondly, the genetic engineering of CAR-T cells to express enzymes that degrade ECM components, such as HPSE, can improve their penetration and homing capabilities in solid tumors. Nonetheless, excessive ECM degradation may compromise the normal architecture of tumor tissues, potentially leading to adverse effects such as bleeding, and may also facilitate the dissemination and metastasis of tumor cells (70). Thirdly, while the overexpression of chemokine receptors has demonstrated some efficacy in augmenting the migratory capacity of CAR-T cells, this approach is not without its limitations. Primarily, the intricate nature of the TME can result in mismatches between chemokines and their receptors, thereby compromising the effectiveness of CAR-T cell therapy (65). Furthermore, the overexpression of chemokine receptors may cause nonspecific cell aggregation in non-target tissues, thereby elevating the risk of adverse side effects (71).

### 3.5 The persistence of CAR-T cells is poor

In the context of breast cancer treatment, CAR-T cells encounter intricate immune regulatory mechanisms upon entering the body, which subsequently impact their persistence. Firstly, breast cancer cells promote immune evasion and accelerate CD8+ T cell exhaustion by upregulating inhibitory molecules (e.g., PD-L1) (72). Secondly, the *in vivo* survival duration of CAR-T cells is constrained, as they progressively differentiate into effector forms with reduced longevity. Specifically, initial stem cell-like (Tn) and central memory T cells (Tcm) transition into effector memory T cells (Tem) (73). Thirdly, the metabolic state or pathway of CAR-T cells significantly influences their persistence. For instance, the TME is characterized by hypoxia and nutrient scarcity, which impair and diminish the normal metabolism of CAR-T cells, ultimately leading to T cell exhaustion and dysfunction (74). Moreover, the differential expression patterns of target antigens between solid tumors, such as breast cancer, and hematological malignancies significantly influence the persistence of CAR-T cells. In breast cancer, common CAR-T cell targets, including HER2, Trop2, and MUC1, are predominantly localized on the surface of tumor cells. Conversely, CD19 CAR-T cells, which represent a well-established treatment for hematological malignancies, target the CD19 antigen expressed on both malignant B cells and normal B lymphocytes. The continuous antigenic stimulation provided by normal B cells facilitates the activation and proliferation of CD19 CAR-T cells, thereby preventing premature exhaustion and promoting sustained long-term anti-tumor activity. This continuous stimulation offers a distinct advantage over CAR-T cells targeting breast cancer, which lack the additional antigenic stimulation from healthy cells or receive only weak stimulatory signals.

#### 3.5.1 Coping strategies

The combination of ICIs and CAR-T cell therapy is one of the important strategies to improve the durability of CAR-T cells. ICIs have been shown to mitigate CAR-T cell depletion by inhibiting immune checkpoint molecules (75, 76). Notably, ICIs are pivotal in addressing the immunosuppressive TME associated with breast cancer. The TME in breast cancer is typically characterized by immunosuppression, which hinders the immune system’s ability to effectively target tumor cells. ICIs mitigate this suppression by inhibiting immune checkpoint proteins on the surfaces of both tumor and immune cells, thereby enhancing the anti-tumor immune response (77). In the context of breast cancer, particularly TNBC, ICIs demonstrate significant therapeutic potential. They improve the immune milieu of the TME by augmenting the number and activity of tumor-infiltrating lymphocytes, consequently enhancing the treatment response rate (78). Furthermore, the application of gene editing techniques, such as CRISPR/Cas9, to disrupt the expression of immune checkpoint molecules can also enhance the durability and functionality of CAR-T cells (79). In addition to ICIs, other pharmacological agents, such as STING agonists and PI3K inhibitors, have been identified as potential enhancers of CAR-T cell efficacy. For instance, Xu et al. (80) demonstrated that the concurrent administration of STING agonists, such as DMXAA, significantly improves the infiltration and persistence of CAR-T cells within the TME, thereby enhancing their anti-tumor activity. This enhancement is likely mediated by the ability of STING agonists to induce the production of chemokines, which facilitates the recruitment of CAR-T cells. Furthermore, PI3K inhibitors are also used to regulate the differentiation process of CAR-T cells and maintain their low differentiation state, thus improving their durability and anti-tumor efficacy (73).

The activity of CAR-T cells derived from initial T cells (Tn) and central memory T cells (Tcm) is superior to that of CAR-T cells derived from effector memory T cells (Tem), demonstrating improved and sustained efficacy (81). Moreover, CAR-T cells *in vivo* gradually differentiate into effector forms with shorter life spans (73). The differentiation process of CAR-T cells can also be regulated by gene editing technology. For instance, the deletion of SUV39H1 can enhance the anti-tumor function of CAR-T cells by increasing the chromatin accessibility of stem cells/memory-related genes, while reducing the appearance of effect/failure phenotype (82).

The metabolic state and pathway of CAR-T cells have an important influence on their persistence. Modulating the metabolic pathways of CAR-T cells can substantially enhance their *in vivo* persistence and antitumor efficacy. Metabolic reprogramming is regarded as a potent strategy to augment the durability of CAR-T cells. Through the regulation of these metabolic pathways, both the persistence and antitumor activity of CAR-T cells *in vivo* can be markedly improved. Firstly, in response to the hypoxic and low-nutrition characteristics of the TME, Niu et al. (74) effectively engineered CAR-T_Foxp3_ cells endowed with metabolic reprogramming capabilities through the co-expression of the Foxp3 gene alongside third-generation CAR constructs. These engineered cells demonstrated diminished aerobic glycolysis and oxidative phosphorylation, coupled with an augmented lipid metabolism. The findings indicated that CAR-T_FOXP3_ cells were capable of sustaining their metabolic function under hypoxic and nutrient-deprived conditions within the TME, thereby averting cell depletion and preserving robust antitumor activity. Secondly, the application of metabolic priming in CAR-T cell production holds considerable promise for augmenting memory phenotype and persistence. By initially activating CAR-T cells in a low glucose/glutamine environment, followed by expansion in a high glucose/glutamine environment, researchers have successfully generated CAR-T cells exhibiting elevated CCR7/CD62L expression and improved persistence. This approach not only mitigates glycolysis but also enriches the central memory phenotype of CAR-T cells, thereby enhancing their persistence *in vivo* (83). Thirdly, metabolic reprogramming has the potential to improve the durability of CAR-T cells through the modulation of specific metabolic pathways. For instance, the addition of D-mannose has been demonstrated to increase the anti-tumor efficacy of T cells and reduce differentiation-induced exhaustion. This is accomplished by promoting intracellular metabolic programming and enhancing the O-GlcNAcylation of β-catenin (84).

#### 3.5.2 The critical assessment of coping strategies

- The integration of ICIs with CAR-T cell therapy has the potential to enhance the persistence and functionality of CAR-T cells by targeting immune checkpoint molecules, such as PD-1/PD-L1. However, this approach may not be universally applicable to all breast cancer patients due to significant interpatient variability in the tumor microenvironment. For instance, the expression levels of immune checkpoint molecules vary among individuals, and the therapeutic efficacy of ICIs is closely associated with these expression levels (85). Furthermore, the use of ICIs may exacerbate treatment-related side effects, such as immune-related adverse events (irAEs), thereby limiting their widespread clinical application.
- The selection of Tn and Tcm cells for the construction of CAR-T cells can extend their persistence *in vivo*; however, maintaining a stable memory phenotype in practical applications remains challenging.
- Metabolic reprogramming represents another strategy to enhance the durability of CAR-T cells. By modulating the metabolic pathways of CAR-T cells, they can better adapt to the hypoxic and nutrient-deficient conditions of the tumor microenvironment. While this approach shows promise in basic research, precisely controlling the extent of metabolic reprogramming in clinical applications to prevent potential aberrant cell functions remains an urgent issue that needs to be addressed.

### 3.6 Toxic reaction

In the context of CAR-T cell therapy for breast cancer, the occurrence of toxic reactions represents a significant concern. Cytokine release syndrome (CRS), neurotoxicity, and “on-target off-tumor effect” are the toxic reactions that have attracted much attention at present. These problems not only affect the safety of treatment but also limit its wide application in clinic.

Firstly, CRS represents the most prevalent adverse reaction associated with CAR-T therapy. The onset of CRS is primarily attributed to the substantial activation of CAR-T cells, resulting in the overproduction of cytokines, including IL-6, TNF-α, and IFN-γ, ultimately precipitating the development of CRS (86, 87). This hyperactive immune response can result in severe clinical manifestations, including fever and hypotension, and may progress to life-threatening conditions (88).

Secondly, neurotoxicity represents a significant concern in CAR-T cell therapy. This condition may present with symptoms such as confusion and seizures (89). While the exact mechanism is not yet fully elucidated, it is hypothesized to be associated with increased permeability of the blood-brain barrier (BBB). This increased permeability is potentially induced by the activation of endothelial cells within the central nervous system, allowing cytokines and CAR-T cells from the bloodstream to infiltrate the cerebrospinal fluid, thereby facilitating the onset of neurotoxic effects (90–93).

Thirdly, on-target off-tumor effect is another challenge to be solved in CAR-T cell therapy (94). This effect is due to the fact that the targets of CAR-T cell therapy for breast cancer are tumor-associated antigens (TAAs) instead of tumor-specific antigens (TSAs). These antigens are expressed relatively high in tumor cells, but there may be low levels of expression in normal tissues. When CAR-T cells are transfused into the human body, they may recognize and attack these normal cells that express low-level antigens, thus triggering a series of adverse reactions (89). One significant event was an early clinical trial involving HER2-CAR-T cells. In this trial (6), a patient with HER2-positive metastatic colorectal cancer received an infusion of HER2-CAR-T cells; however, the patient unfortunately succumbed five days post-treatment. The cause of death was attributed to the localization of CAR-T cells in the lungs, which recognized normal lung epithelial cells expressing low levels of HER2. The recognition led to cytokine release, which triggered a cytokine storm, resulting in acute lung injury and respiratory failure. This incident demonstrated that even low-level HER2 expression in lung tissue can provoke a fatal “on-target off-tumor effect” of CAR-T cells. Consequently, this event has had a profound impact on the field, prompting subsequent research to more rigorously assess target safety, optimize CAR affinity, and explore safer drug delivery strategies.

#### 3.6.1 Coping strategies

The identification of specific tumor antigens is crucial in the development of CAR-T cell therapies. Selecting tumor antigens that exhibit high specificity and low expression in normal tissues as targets can mitigate the risk of on-target off-tumor effects. Furthermore, the pursuit of novel tumor-specific antigens remains a significant avenue for reducing toxicity. This can be achieved by employing genomics, proteomics, and other advanced technologies to screen for additional tumor-specific antigens, thereby expanding the repertoire of potential targets for CAR-T cell therapy.

Optimizing the structure of CARs can reduce the activity of CAR-T cells and decrease the occurrence of toxic reactions. This optimization is achieved through the integration of regulatory elements within the CAR to modulate CAR-T cell activity under specific conditions. For instance, suicide genes like HSV-TK and iCasp9 are incorporated into the CAR. Upon overactivation of CAR-T cells, these suicide genes are triggered, inducing apoptosis in the CAR-T cells and thereby swiftly managing toxic reactions (95, 96). Furthermore, the implementation of logic gate control mechanisms, including AND-gate and NOT-gate CARs, constitutes a pivotal strategy for enhancing therapeutic precision. The AND-gate CAR is designed to activate only upon simultaneous recognition of two specific antigens on the surface of tumor cells, thereby minimizing the risk of attacking cells that express only a single antigen. Conversely, the NOT-gate CAR employs an inhibitory CAR to automatically deactivate CAR-T cells upon encountering normal tissues that express non-tumor markers, thereby selectively sparing normal cells (97). Furthermore, to achieve precise regulation of CAR-T cell activation and prevent excessive immune responses, the ON/OFF-switch CAR incorporates exogenous small-molecule regulatory switches. This enables precise spatio-temporal control over CAR-T cell activity, allowing for the activation or deactivation of its anti-tumor function as required, thus achieving a balance between efficacy and toxicity (98).

The local administration of CAR-T cells can significantly increase their concentration at the tumor site while minimizing systemic distribution, thereby reducing the risk of on-target off-tumor toxicity to healthy tissues. For instance, CAR-T cells may be directly administered to the tumor via intratumoral injection or regional perfusion, thereby increasing the therapeutic concentration at the tumor site while decreasing systemic exposure (99). In a phase 0 clinical trial (11), six patients with metastatic breast cancer (comprising four with TNBC and two with estrogen receptor-positive, HER2-negative cancer) received a single intratumoral injection of c-Met-CAR-T cells (at doses of 3×10^7 or 3×10^8 cells). The findings indicated that the tumor tissue at the injection site exhibited extensive necrosis, loss of c-Met expression, and infiltration by CD4^+^ T cells and CD68^+^ macrophages, suggesting a localized inflammatory response. Furthermore, all patients tolerated the treatment well, with no treatment-related adverse events of grade 2 or higher and no occurrences of CRS.

#### 3.6.2 The critical assessment of coping strategies

Firstly, the selection of TSAs is considered an optimal strategy to mitigate the “on-target off-tumor” effect. Nonetheless, breast cancer typically exhibits a paucity of TSAs in the strictest sense. Most targets, including HER2 and MUC1, are classified as TAAs, which are expressed at low levels in normal tissues. Consequently, the risk of off-target effects cannot be entirely eliminated.

Secondly, the incorporation of regulatory elements, such as suicide genes and logic gate control mechanisms (AND/NOT-gates), into the CAR structure allows for precise modulation of CAR-T cell function, thereby mitigating the incidence of toxicities and adverse effects. Nonetheless, activation of the suicide gene may result in excessive apoptosis of CAR-T cells, thus reducing their anti-tumor efficacy. The AND-gate CAR is designed to activate only upon recognition of two specific antigens, whereas the NOT-gate CAR can deactivate its function upon encountering normal tissue markers. While these designs are highly innovative, their complexity may elevate production costs and technical challenges. Furthermore, although the logic gate system for multi-antigen recognition theoretically enhances the specificity and efficacy of cancer treatment by concurrently identifying multiple tumor antigens, it still confronts challenges related to tumor heterogeneity and antigen loss in practical applications.

### 3.7 The preparation process is complicated and the cost is high

The individualized preparation process of CAR-T cells is intricate, time-intensive, and costly, significantly hindering its widespread application in breast cancer treatment, particularly in developing countries. The preparation of CAR-T cell therapy encompasses numerous complex stages. Initially, the isolation of T cells from patients’ peripheral blood necessitates stringent aseptic techniques and specialized equipment. Subsequently, the construction of an efficient gene vector and the stable integration of the CAR gene into T cells via genetic engineering are essential. During the *in vitro* expansion of CAR-T cells, precise control of culture conditions—such as cell density, medium composition, and culture duration—is crucial to maintain the cells’ viability and functionality.

Secondly, the large-scale production of CAR-T cell therapy is governed by stringent quality control protocols and must adhere rigorously to Good Manufacturing Practice (GMP) standards. Each batch of CAR-T cell products undergoes comprehensive testing, which includes assessments of cell viability, purity, functional activity, endotoxin levels, and genetic stability. These assessments require the utilization of a wide array of testing equipment and methodologies, consequently increasing production costs.

Finally, the individualized nature of CAR-T cell therapy complicates cost dilution, as each patient’s treatment requires the bespoke preparation of CAR-T cell products. Currently, achieving large-scale standardized production remains unattainable, contributing to elevated production costs.

#### 3.7.1 Coping strategies

Non-viral vectors, including transposon systems, mRNA, and nanocarriers, present a cost-effective and practical alternative for the generation of CAR-T cells, thereby reducing the financial burden associated with gene introduction. Transposon systems, such as Sleeping Beauty and piggyBac, have garnered significant attention due to their low cost, high efficiency in gene transfer, and stable integration capabilities. These systems are straightforward to produce, amenable to large-scale production, and contribute to reduced preparation costs (100, 101). Research indicates that CAR-T cells developed using the Sleeping Beauty transposon system exhibit robust anti-tumor activity both *in vitro* and *in vivo*, while also complying with GMP production standards (102, 103). It is important to recognize that, at present, GMP-grade non-viral vectors do not exhibit significant cost advantages over viral vectors. This is largely attributable to the nascent stage of GMP-compliant manufacturing technologies for non-viral vectors. The optimization of processes for large-scale production, such as the purification of transposon plasmids and the quality control of lipid nanoparticles, alongside the fulfillment of stringent regulatory requirements, entails considerable initial expenses. Nevertheless, non-viral vectors hold promise for future cost reductions. As manufacturing technologies advance, exemplified by the automation of mRNA or nanocarrier production, and as production scales increase, the per-unit cost of GMP-grade non-viral vectors is anticipated to decline substantially, thereby addressing the current cost constraints.

mRNA electrotransduction technology is extensively employed in the construction of CAR-T cells due to its simplicity and affordability, making it suitable for short-term therapeutic applications. CAR-mRNA is synthesized through *in vitro* transcription, allowing for a rapid preparation time of only a few hours, and can be mass-produced using standard kits. By optimizing the conditions for electrotransduction, transfection efficiency can reach up to 95%, with a cell viability loss of less than 2% (104). Furthermore, as an emerging non-viral vector, nanocarriers present several advantages, including non-immunogenicity, ease of production, multifunctionality, and cost-effectiveness (105). Their application in the generation of CAR-T cells is primarily evident in their potential as gene delivery tools. For instance, lipid nanoparticles are employed to deliver mRNA and DNA, facilitating the *in vivo* generation of CAR-T cells. This approach enhances the efficiency of gene delivery while mitigating the security risks associated with viral vectors (105, 106).

The automated, fully enclosed, and cGMP-compliant production of CAR-T cells represents not only an effective approach to addressing current manufacturing challenges but also a primary direction for future advancements (107). The imperative for automated CAR-T cell production lies in enhancing production efficiency and reducing costs. Research indicates that large-scale production of CAR-T cells can be achieved using the CliniMACS Prodigy platform, which facilitates automated production, while gene transfer is accomplished via the virus-free Sleeping Beauty transposon system. This approach not only enhances production efficiency but also significantly decreases operator labor time, thereby reducing production costs (108). Furthermore, another study introduced an innovative semi-automated 24-hour CAR-T cell production process, capable of generating CAR-T cells with high cytotoxicity and cytokine release capabilities within a short timeframe, further demonstrating the feasibility and advantages of automated production (109).

The development of universal CAR-T cells aims to facilitate large-scale production and reduce preparation costs. Traditional autologous CAR-T therapy requires customization for each patient, resulting in high costs and prolonged production timelines. Conversely, allogeneic CAR-T cells are derived from the T cells of healthy donors, with gene editing employed to remove endogenous TCR and HLA-related genes. This genetic modification mitigates the risk of immune rejection and graft-versus-host disease (GVHD) following the infusion of allogeneic CAR-T cells. Consequently, “universal cell products” can be mass-produced from healthy donors and administered to multiple patients.

**Figure 3:**
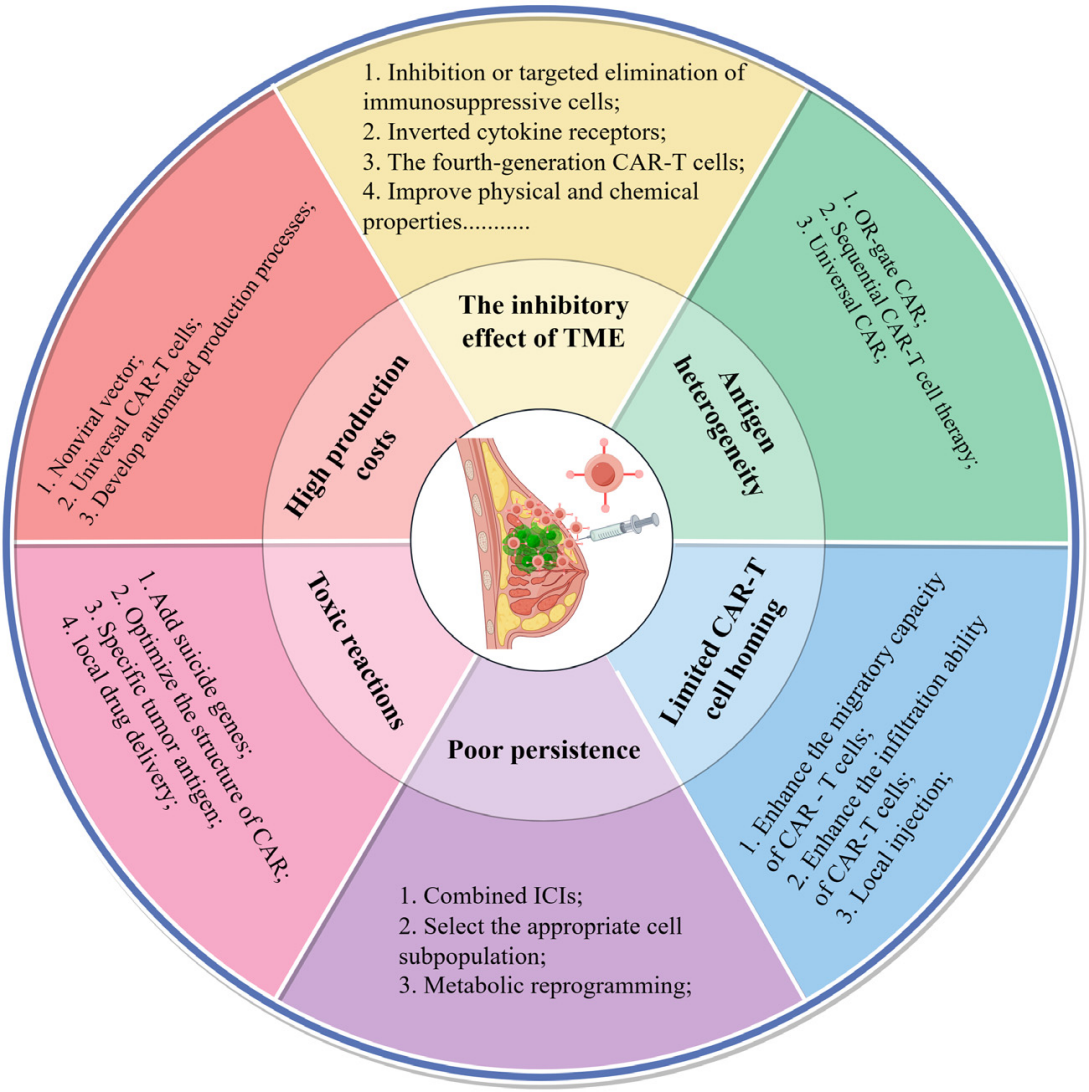
Six key challenges and coping strategies of CAR-T cells in the treatment of breast cancer. The second concentric circle delineates the primary challenges encountered in this area of research, while the outermost circle details the strategies devised to address these challenges. This figure was drawn by Figdraw (a drawing platform).

In conclusion, CAR-T cell therapy encounters several significant challenges in the treatment of breast cancer, including immunosuppression within the tumor microenvironment, antigen heterogeneity, difficulties in homing, limited persistence, potential toxic reactions, and high associated costs. In response to these challenges, researchers have proposed a variety of innovative strategies aimed at addressing these issues. To elucidate the relationship between these challenges and their corresponding solutions, **Figure 3** provides a systematic summary of the key obstacles faced by CAR-T cell therapy in breast cancer and the targeted strategies developed to overcome them, thereby facilitating a comprehensive understanding of the primary research directions in this field.

### 3.8 Facing the competition from other immunotherapies

The advancement of CAR-T cell therapy in the realm of breast cancer treatment is not occurring in isolation; it is encountering significant competition from other emerging immunotherapies. These alternative approaches demonstrate potential advantages in terms of ease of transformation, safety, or specificity compared to CAR-T cell therapy in certain aspects, thereby introducing uncertainty regarding the clinical outlook of CAR-T cell therapy.

Bispecific antibodies (BsAbs) represent a formidable alternative to CAR-T cell therapy. They can closely connect T cells with tumor cells through “bridging” effect, thus activating the cytotoxic effect mediated by T cells. Compared to CAR-T therapy, BsAbs offer several distinct advantages: firstly, they are available as off-the-shelf therapeutics, obviating the need for complex individualized cell preparation processes and reducing associated costs (110). Secondly, BsAbs possess a relatively short half-life, allowing for rapid cessation of treatment in the event of severe toxic reactions by simply discontinuing the drug (110, 111). In the context of HER2-positive breast cancer treatment, BsAbs have demonstrated efficacy in inhibiting tumor growth and enhancing immune-mediated tumor clearance by targeting HER2 in conjunction with other molecules, such as PD-1/PD-L1 (112, 113). This combinatorial approach not only facilitates the direct eradication of HER2-positive tumor cells but also augments anti-tumor activity through antibody-dependent cell-mediated cytotoxicity (ADCC) (112).

NK cell-based therapy represents a rapidly advancing area of research. Chimeric antigen receptor Natural Killer (CAR-NK) cells integrate the targeting specificity of CAR technology with the intrinsic anti-tumor properties of NK cells. Compared to CAR-T cells, CAR-NK cells typically induce either no or only mild CRS and neurotoxicity, thereby offering a superior safety profile (114). Furthermore, NK cells do not elicit graft-versus-host disease (GVHD), allowing them to be effectively sourced from allogeneic donors, such as umbilical cord blood, the NK-92 cell line, or induced pluripotent stem cells. This capability facilitates “off-the-shelf” mass production, addressing the cost and time constraints associated with the personalized nature of CAR-T therapy (115). Additionally, unmodified NK cell adoptive therapy, in conjunction with antibodies that target NK cell activation receptors (e.g., anti-NKG2D antibodies), can further augment the cytotoxic potential of NK cells (116–118).

T cell receptor-engineered T (TCR-T) cell therapy offers a distinct targeting approach compared to CAR-T cell therapy. While CAR-T cells are limited to recognizing tumor-associated antigens expressed on the cell surface, TCR-T cells have the capability to identify intracellular antigens presented by the major histocompatibility complex (MHC). This ability significantly broadens the spectrum of targetable antigens, particularly for tumors with few surface antigens but many mutations that result in new antigens (119).

CAR-macrophage (CAR-M) therapy represents an innovative frontier in biotechnology, offering a novel approach to addressing the challenge of inadequate infiltration of CAR-T cells in solid tumors. Macrophages, as immune cells inherently present within the tumor microenvironment (TME), possess robust phagocytic and antigen-presenting capabilities. CAR-M therapy not only facilitates the direct engulfment of tumor cells but also has the potential to remodel the TME by converting immunosuppressive M2-type macrophages into pro-inflammatory M1-type macrophages. Additionally, it can recruit and activate other endogenous immune cells, potentially leading to a more sustained anti-tumor immune response (120–122).

The future success of CAR-T cell therapy hinges not only on technological advancements—such as overcoming the tumor microenvironment, enhancing durability, and managing toxicity—but also on demonstrating unique competitive advantages over emerging therapies in efficacy, safety, production convenience, and cost-effectiveness. Future clinical research and development strategies should prioritize exploring synergistic combinations between CAR-T cells and these therapies, rather than engaging in direct competition.

### 3.9 The gap from laboratory to sickbed: an analysis of the disconnection between maturity and transformation of coping strategies

#### 3.9.1 The comparative analysis of coping strategies

Although various strategies for addressing the challenges associated with CAR-T cell therapy in breast cancer have been discussed previously, it is imperative to assess their clinical maturity and translational potential. **Table 3** provides a summary of this comparative analysis, categorizing the current strategies, representative approaches, stages of development, core advantages, significant limitations, and primary barriers to clinical translation.

**Table 3:** Comparative analysis of key strategies for CAR-T cell therapy in breast cancer treatment.

Strategies	Representative approaches	Current stages	Key advantages	Major risks	Translational challenges
Targeting TME	inverted cytokine receptor (ICR)	Preclinical	Converts inhibitory cytokine signals into stimulatory signals (54)	May cause over-activation and uncontrolled proliferation of CAR-T cells *in vivo*, triggering immune-related adverse events (irAEs)	Safety and efficacy in the complex human TME require validation
	Combination with TME-modulating drugs (e.g., PARP inhibitors)	Preclinical	Enhances the anti-tumor function of CAR-T cells by specifically inhibiting the recruitment of MDSCs (43)	Both PARP inhibitors and CAR-T cell therapy can cause side effects like hematological toxicity; combination may increase this risk (123)	Determining the optimal timing and dosage for combining PARP inhibitors with CAR-T cells is crucial for efficacy, yet no standard protocol exists
Overcoming Antigen Heterogeneity	Tandem CAR-T Cells	Preclinical	Reduces the risk of immune escape due to loss or downregulation of a single antigen	May broaden the scope of “off-target” toxicity in normal tissues	Ensuring stable, efficient, and consistent production of tandem CAR constructs is more challenging than for single-target CAR-T cells, demanding higher standards in manufacturing and quality control
Enhancing the homing ability of CAR-T cells	Local/Regional Delivery	Phase 1 (e.g., NCT03696030, NCT05623488)	Significantly increases local concentration of CAR-T cells; reduces systemic toxicity reactions (66)	Intratumoral injection may cause tumor rupture, bleeding, or puncture-site infection	Delivery methods (injection depth, dose, frequency) vary greatly for different tumor sites (e.g., primary breast vs. brain metastases), lacking standardized protocols; heavily reliant on precise imaging guidance, requiring specialized equipment and expertise
Improving Persistence	Combination with Immune Checkpoint Inhibitors (ICIs)	Phase 1/2 (e.g., NCT02414269)	ICIs block inhibitory signals (e.g., PD-1) on CAR-T cells, reversing their functional exhaustion and restoring their killing activity and proliferative capacity (75, 76)	ICIs may increase treatment side effects, such as immune-related adverse events (irAEs)	Treatment regimens need optimization, requiring extensive clinical exploration; both therapies are expensive, and combination significantly increases the economic burden
Improving Safety	ON/OFF-switch CAR	Phase 1/2 (e.g., NCT04650451)	Provides precise spatiotemporal control, greatly enhancing safety (98)	Risk of switch failure, leading to efficacy or safety issues	Complex manufacturing process; ensuring the absolute reliability and ease of clinical management of the switch system
	Suicide Gene	Preclinical	When CAR-T cells are overactivated, these suicide genes are triggered, inducing apoptosis and rapidly controlling toxic reactions (95, 96)	Activation of the suicide gene may lead to excessive apoptosis of CAR-T cells, thereby weakening their anti-tumor effect	A balance must be struck between ensuring the safety of CAR-T cells and preserving their therapeutic efficacy

#### 3.9.2 There is a discrepancy between the results of preclinical studies and the actual clinical efficacy

Numerous promising preclinical findings failed to bring significant advantages in the actual clinical treatment of breast cancer. This discrepancy can be attributed to a disconnect between preclinical research outcomes and their actual clinical efficacy. Two primary factors may account for this phenomenon:

1. The High Complexity of the Human TME. Conventional immunodeficient mouse models exhibit significant limitations in replicating the human breast cancer TME. These models typically lack a fully functional human immune system and cannot fully recapitulate the complex immunosuppressive network and stromal density of the human TME (124). Consequently, these models fail to accurately and authentically reflect the immunosuppressive characteristics of human tumors (125, 126). Although humanized mouse models have partially mitigated this issue, they still cannot fully reproduce the complexity of the human TME (125). As a result, preclinical experimental outcomes are not always fully replicable in clinical trials.
2. In preclinical studies, researchers typically utilize breast cancer cell lines characterized by highly uniform and high antigen expression, and administer CAR-T cells directly viaintratumoral or intravenous injection into immunodeficient mice at an optimal effector-to-target ratio. This idealized model of “high antigen density in an immunologically non-interfering environment” significantly amplifies the cytotoxic efficacy of CAR-T cells. However, real-world breast cancers exhibit marked antigen heterogeneity. Upon administration into patients, CAR-T cells face a far more complex reality: they must penetrate the stromal barrier, overcome immunosuppression, and function effectively under conditions where antigen density approaches physiological thresholds. Consequently, the therapeutic efficacy of CAR-T cells in clinical applications is significantly lower than that observed in preclinical experiments.

## 4 Lessons from clinical trials: determinants of response and efficacy limitations

The adaptation of CAR-T cell therapy from hematological malignancies to solid tumors, including breast cancer, has faced significant clinical challenges. While preliminary studies have demonstrated the safety and feasibility of this approach in treating breast cancer, the overall therapeutic efficacy remains moderate. This section synthesizes the previous content and provides an analysis of the factors that distinguish responders from nonresponders, as well as the reasons for the limited efficacy achieved in early trials.

### 4.1 Main factors that distinguish responders from non-responders

The expression level of the target antigen on tumor cells is one of the key factors affecting the curative effect of CAR-T cells (127, 128). Tumor patients with a higher expression level of the target antigen may have a more positive and sensitive response to CAR-T cells. This is because CAR-T cells can identify and target tumor cells with high expression of the target antigen more easily. For instance, CAR-T cells targeting folate receptor α (FRα) showed stronger anti-tumor activity in the TNBC model with a higher expression level of FRα (129). On the other hand, the tumor with low expression of the target antigen has insufficient binding frequency with the CAR protein, which leads to the inability to effectively activate CAR-T cells. In clinical treatment, some patients have drug resistance or relapse after receiving CAR-T cells, which is often related to tumor cells down-regulating or losing the target antigens to escape CAR-T recognition (130). Consequently, precise assessment of tumor target antigen expression levels prior to treatment is of significant clinical importance for selecting appropriate patients, predicting therapeutic outcomes, and optimizing CAR-T treatment strategies.

In the context of CAR-T therapy for solid tumors, the immunosuppressive properties of the TME are another important factor that distinguishes responders from non-responders. The TME in responders may exhibit characteristics of an “immune permissive” or “hot tumor.” These responders often display weaker immunosuppressive signals within the TME, such as reduced levels of PD-L1 and a lower presence of immunosuppressive cells, thereby facilitating the sustained function of CAR-T cells (131, 132). Conversely, the TME in non-responders is often characterized as a “cold tumor” environment. This environment is marked by intense metabolic competition, including hypoxia and nutrient deprivation, and a robust immunosuppressive network, all of which contribute to the depletion, dysfunction, and impaired infiltration of CAR-T cells (72, 133, 134). Furthermore, the overexpression of immune checkpoint molecules, such as PD-L1, by the TME in non-responders can exacerbate CAR-T cell depletion (72).

### 4.2 Underlying reasons for modest efficacy in early clinical trials

Firstly, early clinical trials predominantly utilized second-generation CAR-T cells. However, these CAR-T cells demonstrated limited efficacy in overcoming the challenges posed by tumor microenvironment inhibition, cell homing disorders, and poor *in vivo* persistence. Consequently, their anti-tumor effects were restricted.

Secondly, the design of early clinical trials exhibits several limitations. For instance, the optimal dosing of CAR-T cells has not been thoroughly investigated. The majority of trials refrained from conducting dose escalation studies due to concerns about potential toxicities, such as CRS. Furthermore, no combination therapy strategies were implemented. As previously noted, combining CAR-T therapy with ICIs or PARP inhibitors has the potential to enhance therapeutic efficacy. However, the early trials exclusively employed CAR-T monotherapy, which did not achieve improved therapeutic outcomes through combination approaches.

Thirdly, the sample size in these studies is typically limited. The majority of initial clinical trials in this domain are in the exploratory phases 0/I, characterized by small sample sizes. This limitation may result in inadequate statistical power, making it difficult to exclude the influence of random errors on the outcomes. Additionally, the restricted sample size constrains the investigation of dose-efficacy and toxicity-efficacy relationships in clinical experiments, thereby exacerbating the adverse impact of design deficiencies, such as inadequate dose exploration.

Finally, adopting a multi-target strategy or logic gate design (such as OR-gate) can expand the recognition range of CAR-T cells. However, early CAR-T cells were designed with a single target, which was easily influenced by tumor antigen heterogeneity and tumor antigen down-regulation/loss, leading to antigen escape.

## 5 Conclusion

In the field of breast cancer treatment, CAR-T cell therapy presents promising potential due to its unique advantages. Nevertheless, its clinical application is hindered by several challenges, including the immunosuppressive characteristics of the tumor microenvironment, antigen heterogeneity, obstacles in CAR-T cell homing, limited persistence, and potential toxic reactions. These challenges limit the efficacy and wider adoption of CAR-T cells in breast cancer therapy.

This paper provides a comprehensive overview of the primary targets and the preclinical and clinical advancements of CAR-T cell therapy in the treatment for breast cancer. It also offers a critical analysis of the associated challenges and the strategies devised to address them. Notably, the transition from basic research to clinical application necessitates prioritizing several key clinical translation points. Firstly, strategies to overcome the immunosuppressive TME should be implemented in clinical practice. For instance, CAR-T cells can be combined with drugs that modulate the TME, such as ICIs or PARP inhibitors, to enhance anti-tumor efficacy. Secondly, promoting the clinical application of safety-enhanced CAR designs, such as AND gate/NOT gate logic control systems and suicide genes, is essential, along with accumulating long-term clinical data in phase II/III trials. Thirdly, there is a need to advance the industrialization of non-viral vectors and automated, closed GMP-compliant production platforms to reduce costs and shorten production cycles. Future research directions in this field may encompass several key areas. Firstly, elucidating the molecular mechanisms underlying metabolic reprogramming to enhance the durability and functionality of CAR-T cells is crucial. Secondly, designing reasonable clinical trials for combination therapies, such as CAR-T with ICIs or PARP inhibitors, is essential to validate their synergistic efficacy and safety. Thirdly, further optimization of CAR structures or integration with emerging technologies, such as nanotechnology, is necessary to improve the durability, anti-tumor activity, and safety of CAR-T cells. Lastly, advancing the development of “off-the-shelf” allogeneic CAR-T products is important for cost reduction.

In conclusion, while CAR-T cell therapy for breast cancer remains in the exploratory phase of clinical translation, its potential is progressively being realized through ongoing advancements in target recognition, tumor microenvironment regulation, production process optimization, and safety control. By prioritizing these aspects of clinical translation, the transition of CAR-T cell therapy from fundamental research to clinical practice is anticipated to accelerate, ultimately offering more effective treatment options for breast cancer patients globally.
